# Acquired Antibody Responses against *Plasmodium vivax* Infection Vary with Host Genotype for Duffy Antigen Receptor for Chemokines (DARC)

**DOI:** 10.1371/journal.pone.0011437

**Published:** 2010-07-15

**Authors:** Amanda Maestre, Carlos Muskus, Victoria Duque, Olga Agudelo, Pu Liu, Akihide Takagi, Francis B. Ntumngia, John H. Adams, Kim Lee Sim, Stephen L. Hoffman, Giampietro Corradin, Ivan D. Velez, Ruobing Wang

**Affiliations:** 1 Grupo Salud y Comunidad, Facultad de Medicina, Universidad de Antioquia, Medellín, Colombia; 2 Programa de Estudio y Control de Enfermedades Tropicales (PECET), Facultad de Medicina, Universidad de Antioquia, Medellín, Colombia; 3 Seattle Biomedical Research Institute (SBRI), Seattle, Washington, United States of America; 4 University of South Florida, Tampa, Florida, United States of America; 5 Protein Potential LLC., Rockville, Maryland, United States of America; 6 Sanaria, Rockville, Maryland, United States of America; 7 Department of Biochemistry, Université de Lausanne, Lausanne, Switzerland; University of Toronto, Canada

## Abstract

**Background:**

Polymorphism of the Duffy Antigen Receptor for Chemokines (DARC) is associated with susceptibility to and the severity of *Plasmodium vivax* malaria in humans. *P. vivax* uses DARC to invade erythrocytes. Individuals lacking DARC are ‘resistant’ to *P. vivax* erythrocytic infection. However, susceptibility to *P. vivax* in DARC+ individuals is reported to vary between specific DARC genotypes. We hypothesized that the natural acquisition of antibodies to *P. vivax* blood stages may vary with the host genotype and the level of DARC expression. Furthermore, high parasitemia has been reported to effect the acquisition of immunity against pre-erythrocytic parasites. We investigated the correlation between host DARC genotypes and the frequency and magnitude of antibodies against *P. vivax* erythrocytic stage antigens.

**Methodology/Findings:**

We assessed the frequencies and magnitudes of antibody responses against *P. vivax* and *P. falciparum* sporozoite and erythrocytic antigens in Colombian donors from malaria-endemic regions. The frequency and level of naturally-acquired antibodies against the *P. vivax* erythrocytic antigens merozoite surface protein 1 (PvMSP1) and Duffy binding protein (PvDBP) varied with the host DARC genotypes. Donors with one negative allele (FY*B/FY*Bnull and FY*A/FY*Bnull) were more likely to have anti-PvMSP1 and anti-PvDBP antibodies than those with two positive alleles (FY*B/FY*B and FY*A/FY*B). The lower IgG3 and IgG1 components of the total IgG response may account for the decreased responses to *P. vivax* erythrocytic antigens with FY*A/FY*B and FY*B/FY*B genotypes. No such association was detected with *P. falciparum* erythrocytic antigens, which does not use DARC for erythrocyte invasion.

**Conclusion/Significance:**

Individuals with higher DARC expression, which is associated with higher susceptibility to *P. vivax* infection, exhibited low frequencies and magnitudes of *P. vivax* blood-stage specific antibody responses. This may indicate that one of the primary mechanisms by which *P. vivax* evades host immunity is through DARC indirectly down-regulating humoral responses against erythrocytic invasion and development.

## Introduction

Malaria remains the most important parasitic infection in the world with almost half a billion clinical cases every year [1]. It is caused by infection with one or more of five species of *Plasmodium* parasites. However, two species, *P. falciparum* and *P. vivax*, are responsible for most of the morbidity and mortality due to malaria [2], [3], [4], [5], [6]. *P. vivax* malaria does not attract as much attention from the scientific community, government entities or funding agencies as does the more deadly *P. falciparum* malaria. This is partly because *P. vivax* malaria was in the past erroneously referred to as ‘benign’ tertian malaria. But recent studies have revealed that vivax malaria can potentially lead to severe debilitating complications and about 2 billion people spread across 3 continents are continuously at risk of the infection [3], [4], [5], [6], [7], [8]. The discovery and development of novel interventions, most especially vaccines, will depend upon a better understanding of parasite biology and the naturally induced immune response in humans [9], [10], [11], [12].

An important biological difference between *P. vivax* and *P. falciparum* is that only *P. vivax* merozoites use the Duffy (Fy) antigen/receptor for chemokines (DARC) to invade erythrocytes [13], [14]. DARC is a glycosylated membrane protein that is encoded by a gene located on the long arm of chromosome 1 [15], [16], [17], [18], [19]. DARC is more abundant on the surface of reticulocytes than on mature erythrocytes and is also expressed on the endothelial surfaces of some organs [17], [18]. DARC binds to most inflammatory chemokines and its roles in the immune system include leukocyte activation and recruitment [20], [21]. In addition, DARC is associated with protection and susceptibility to a number of other infectious and non-infectious diseases [22], [23], [24], [25], potentially via its elimination of excess toxic chemokines produced during infectious processes and its regulation of leukocyte trafficking [26], [27]. Finally, DARC is the essential receptor required for the entry of *P. vivax* into erythrocytes [13].

Genetic polymophisms have been identified in humans that affect the expression of the Duffy antigen and the susceptibility to blood stage infection by *P. vivax*. Fy+ individuals have 5 common genotypes (FY*A/FY*A, FY*A/FY*B, FY*A/FY*Bnull, FY*B/FY*B and FY*B/FY*Bnull) that produce 3 major phenotypes (Fy a^+^/b^+^, Fy a^+^/b^−^, Fy a^−^/b^+^). Red cells from all Fy+ individuals are susceptible to *P. vivax* infection. People with mutations in the DARC promoter region that abolish DARC expression (FY*Bnull/FY*Bnull or FY*O) exhibit a Duffy-negative phenotype (Fy−) [28], [29], [30]. Erythrocytes of Fy− individuals cannot be invaded by *P. vivax* merozoites such that Fy− individuals are refractory to *P. vivax* blood stage infection. However, since sporozoite invasion of hepatocytes does not involve DARC, both Fy+ and Fy− individuals are susceptible to infection by sporozoites and develop liver stage parasites. But upon release of liver merozoites into the circulation, only Fy+ individuals develop blood stage infections because the erythrocytes of Fy(−) individuals do not express DARC required for erythrocyte invasion by merozoites.

The requirement for DARC in erythrocytic vivax infection limits the infection to the liver stage in Fy− individuals. However, both humoral and cellular immune responses against *P. vivax* pre-erythrocytic parasites have been reported in Fy− humans [31]. The frequencies of antibody responses to the *P. vivax* circumsporozoite protein have in fact been found to be similar between Fy− and Fy+ groups. Nevetheless, responses against erythrocytic antigens were significantly less frequent among Fy− individuals than in Fy+ individuals since Fy− individuals do not develop erythrocytic infections [31], [32].

Naturally acquired antibody and T cell responses play a major role in reducing the risk of infection and in clinical protection against malaria infection [33], [34], [35]. Blood stage infections in rodent malaria and in human *P. falciparum* infections have been reported to suppress T cell responses against liver stage antigens [36], [37], [38], [39]. However, suppression of blood stage infections can permit development of immune responses against liver stage parasites, as shown by the discovery of two promising *P. falciparum* liver stage antigens using sera from people exposed to *P. falciparum* malaria while under chloroquine prophylaxis that eliminated blood stage parasites [15], [40], [41]. All these data support the notion that a lower parasite load in the blood may be associated with increased acquired immunity against reinfection and clinical protection.

The development of vaccine(s) and other novel interventions targeted at the control of parasites, especially in this case *P. vivax*, requires a better understanding of naturally-acquired immune response. In previous studies, the cellular and humoral immune responses against vivax antigens among Fy+ donors had been compared to those of Fy− donors [31], [32]. However, the Fy+ phenotype is conferred by 5 different genotypes, and the diversity of immune responses to *P. vivax* exposure in humans with different genotypes has not yet been assessed. Since the degree of DARC expression varies remarkably across different FY genotypes [17], we hypothesized that the acquisition of humoral immunity against the blood stage parasites will also vary with the level of DARC expression. This would be in concurrence with the observation that susceptibility and resistance to the infection varies between specific genotypes [42], [43]. Furthermore, functional interaction of the different alleles of DARC with DBP for erythrocytic invasion, and the different signaling characteristics of the different DARC alleles after they have bound the DBP, may also be associated with the load of *P. vivax* parasites in the blood. Finally, as a chemokine receptor, DARC may also be involved in the immumodulation of innate and acquired immune responses against vivax infection. We therefore embarked on the evaluation of the antibody responses associated with different DARC genotypes.

## Results

DARC is required for the blood stage infection of vivax malaria and its level of expression varies with specific genotypes associated with susceptibility and resistance to *P. vivax* erythrocyte infection [42], [44], [45]. This led us to hypothesize that the acquired antibody responses may also vary with the level of DARC expression. This project is an attempt to increase our knowledge of natural acquired immunity against erythrocytic parasites associated with differential DARC gene expression.

First, we demonstrated differences in the antibody responses to erythroctyic antigens between the Fy+ and Fy− groups. We then demonstrated the potential association of antibody resposnes with different levels of DARC expression [(A/A or A/B)>(A/Bnull or B/B or B/Bnull)] among Fy+ groups. Finally, we wanted to know if the differential antibody responses were associated with not only stratified DARC expression but also with specific DARC genotypes, since it has been reported that susceptibility to erythrocytic vivax infection was different between FY*A and FY*B genotypes [44]. Therefore, we compared the antibody responses between homozygous and heterozygous FYA or FYB genotypes. As controls, we carried out similar evaluations with *P. falciparum* sporozoite and blood stage antigens, which do not require interaction with DARC. The circumsporozoite protein (CSP) was selected as the representative antigen for the pre-erythrocytic stage as it is the immunodominant surface antigen on the sporozoite [46] and also the most advanced vaccine candidates for both vivax and falciparum malaria [47], [48]. Merozoite surface protein 1 (MSP1) was also selected as the representative antigen for the erythrocytic stage because of the its expression on the surface of merozoites that invade new red blood cells [49] and its prominence in both vivax and falciparum vaccine development [50], [51]. The *P. vivax* Duffy Binding Protein (PvDBP) was identified as the parasite protein that engages DARC present on the surface of red blood cells [52], [53] and is also being developed as a vaccine candidate [12].

### Distribution of Duffy genotypes

In Colombia and throughout the South American continent, the majority of the clinical cases of malaria are caused by *P. vivax* (∼70%), while *P. falciparum* is responsible for the remaining ∼30% [54], [55]. We recruited 233 donors from the malaria-endemic regions of Apartardo and Turbo along the Caribbean coast of Colombia (**Table 1**). All donors who participated in this study had been living in the endemic areas for at least 5 years and had not experienced symptomatic malaria in the previous 12 months. About a quarter of the subjects (26%, n = 60) were Duffy negative (FY*Bnull/FY*Bnull). Among the Duffy positive individuals, the heterozygous FY*A/FY*B genotype was the most common genotype in these regions (22%, n = 52). The frequencies of the other genotypes in the study population (FY*A/FY*A, FY*A/FY*Bnull, FY*B/FY*B and FY*B/FY*Bnull) were 9, 13, 15, and 15%, respectively (**Table 1**). The overall prevalence of FY*A and FY*B alleles expressed as homozygous or heterozygous among the entire study population was 44% and 65% respectively. We also recruited 30 donors from Medellin, a non-malaria endemic city in Colombia. These included 5 Fy− (17%) and 25 Fy+ (83%) individuals. Their blood samples were used to standardize the assay protocols and as unexposed controls.

**Table 1:** Frequencies of genotypes for Duffy antigen receptor for chemokines (DARC) under the study.

Endemic areas, Colombia	Genotype frequencies of Duffy positive FY+					Total FY+	Total FY−
	FY*A/*A	FY*A/*B	FY*A/*Bnull	FY*B/*B	FY*B/*Bnull		FY*Bnull/*Bnull
Turbo (N = 155)	13 (8.4)^a^	26 (16.8)	23 (14.8)	17 (11.0)	24 (15.5)	103 (66.5)	52 (33.5)
Apartado (N = 78)	8 (10.3)	26 (33.3)	7 (9.0)	17 (21.8)	12 (15.4)	70 (89.7)	8 (10.3)
Total (N = 233)	21 (9.0)	52 (22.3)	30 (12.9)	34 (14.6)	36 (15.5)	173 (74.2)	60 (25.8)

a Number (%) of individuals expressing the genotype(s).

### Broad antibody responses against *P. vivax* and *P. falciparum* malaria were induced by natural exposure

**Figure 1:**
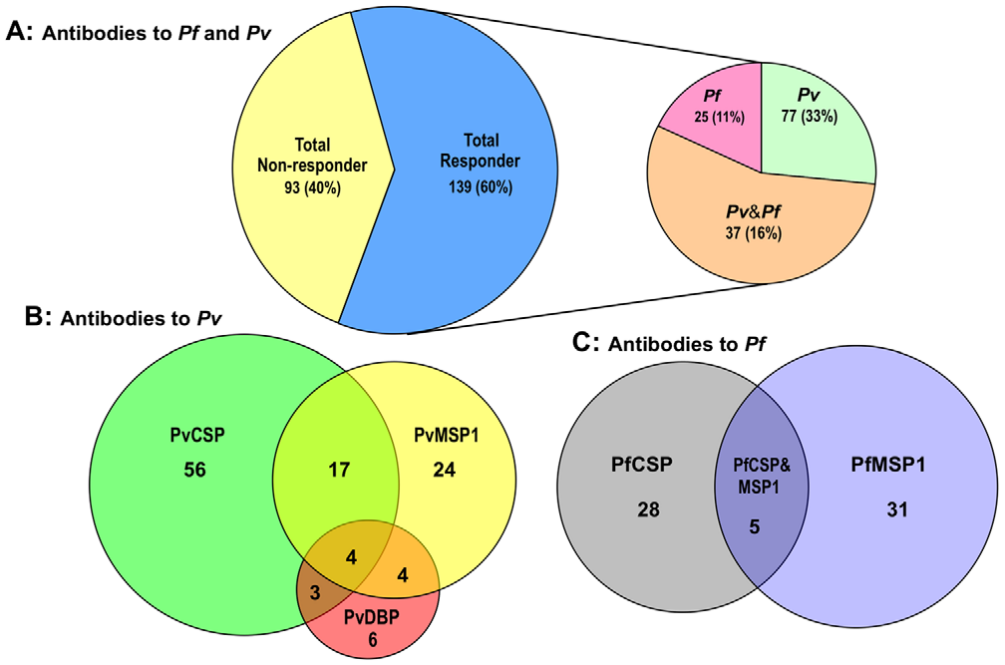
Broad antibody responses against malaria were induced by natural exposures in Colombia. We assessed malaria antigen-specific antibody responses in 233 donors living in Caribbean coast town (Apartado and Turbo) of Colombia. One hundred thirty nine (60%) donors had antibodies against at least one of five malaria antigens examined. Numbers represent the responders who had antibodies against both *P. vivax* and/or *P. falciparum* antigens (A), different *P. vivax* antigens, PvCSP, PvMSP1, PvDBP (B), or different *P. falciparum* antigens PfCSP and PfMSP1 (C).

We assessed antigen-specific antibody responses by ELISA to each of 3 *P. vivax* and 2 *P. falciparum* antigens in sera from each of the individual donors among different DARC genotypes. There were no detectible anti-malaria antibodies in any of the 30 non-exposed control donors living in the non-endemic area (data not shown). The frequency of antibody responses to parasite antigens among donors living in the malaria endemic areas of Apartado and Turbo is presented in **Figure 1**. One hundred thirty-nine donors (60%) had antibodies against at least one of the five *P. vivax* or *P. falciparum* antigens that were tested. Thirty-seven donors (16%) had antibodies against at least one *P. vivax* and one *P. falciparum* antigen (**Figure 1A**). The majority of these (84%, n = 31) were Duffy positive (Fy+) individuals.

A total of 114 donors (49%) had antibodies against at least one of the *P. vivax* antigens assessed (**Figure 1B**). Among these, 28 donors (12%) had antibodies against both *P. vivax* sporozoite [circumsporozoite protein (PvCSP)] and erythrocytic antigens [merozoite surface protein 1 (PvMSP1) or Duffy binding protein (PvDBP)]. Fifty-six donors had antibody against PvCSP. However, only 4 Individuals had antibodies against all 3 *P. vivax* antigens tested (PvCSP, PvMSP1 and PvDBP), while 4 had antibodies against two erythrocytic antigens (PvMSP1 and PvDBP) but not to the sporozoite antigen PvCSP (**Figure 1B**).

Antibody responses to *P. falciparum* antigens are presented in **Figure 1C**. Sixty-four (64) donors (27%) had antibodies against at least one of the two *P. falciparum* antigens tested and 5 donors (2%) had antibodies against both PfCSP and PfMSP1.

The magnitudes of the antibody responses against each antigen are presented as the average Index of Reactivity (IR). The IR is calculated as the average OD value of each sample at 450 nm divided by the sum of average OD values of negative controls (US donors) and 3 standard deviations. Using this standardized IR calculation, none of the 30 donors from the non-malaria endemic area had detectable antibodies to any of the 5 malaria antigens (data not shown). Among the responders from the malaria endemic responders, the average IR for PvCSP, PvMSP1 and PvDBP were 0.83, 0.82, and 0.64, respectively. Those for PfCSP and PfMSP1 were 0.62 and 0.93, respectively (**Table 2**).

**Table 2:** Immune recognition of different *P. vivax* and *P. falciparum* stage-specific antigens.

	Anti-PvCSP			Anti-PvMSP1			Anti-PvDBP			Anti-PfCSP			Anti-PfMSP1		
Fy Genotype	IR Mean±SD	#Pos (%)	*P* value	IR Mean±SD	#Pos (%)	*P* value	IR Mean±SD	#Pos (%)	*P* value	IR Mean±SD	#Pos (%)	*P* value	IR Mean±SD	#Pos (%)	*P* value
A/A	0.68±0.65	4 (19%)	N/A	0.78±0.59	3 (14%)	N/A	0.63±0.25	1 (6%)	N/A	0.69±0.56	4 (19%)	N/A	1.29±0.40	4 (19%)	N/A
A/B	0.94±0.69	19 (37%)		1.16±0.82	13 (25%)		0.67±0.31	2 (4%)		0.68±0.41	11 (21%)		0.94±0.34	10 (19%)	
A/Bnull	1.17±0.75	13 (43%)	0.316	1.45±0.56	13 (43%)	0.086	0.88±0.42	4 (22%)	0.025	0.66±0.47	4 (14%)	0.453	1.14±0.51	7 (23%)	0.659
B/B	0.70±0.53	9 (26%)		0.57±0.43	3 (9%)		0.55±0.21	2 (6%)		0.66±0.48	4 (12%)		0.99±0.27	5 (15%)	
B/Bnull	0.90±0.56	15 (42%)	0.181	1.08±0.58	12 (33%)	0.012	1.04±0.84	8 (27%)	0.022	0.63±0.32	4 (11%)	0.932	0.69±0.25	4 (11%)	0.653
Total Fy+	0.88±0.64	60 (35%)		1.01±0.60	44 (16%)		0.80±0.45	17 (12%)		0.66±0.38	27 (16%)		1.01±0.33	30 (17%)	
Bnull/Bnull	0.79±0.58	20 (33%)	0.850	0.64±0.39	5 (8%)	0.005	0.54±0.18	0 (0%)	0.001	0.57±0.39	6 (10%)	0.308	0.84±0.22	6 (10%)	0.175

Antigen-specific total IgG in sera of the donors living in malaria-endemic regions of Colombia (Apartado and Turbo) were assessed by ELISA against *P. vivax* circumsporozite protein (PvCSP), blood stage antigens (PvMSP1, *Pv*DBP), *P. falciparum* circumsporozoite protein (PfCSP) and blood stage antigen (PfMSP1). A sample is considered positive if the index of reactivity (IR) is greater or equal to 1. IR is calculated as the average OD of sample divided by the cut-off OD with the cut of OD being the sum of average OD values of US donors (with no prior exposure to malaria) + 3 standard deviations. Listed are the mean and SD of the IR, the number and percentage of positive samples (#Pos (%)), *P value*s were caulcated by Chi-squared test.

### Antibodies against *P. vivax* pre-erythrocytic antigen are comparable among Fy− and Fy+ donors

**Figure 2:**
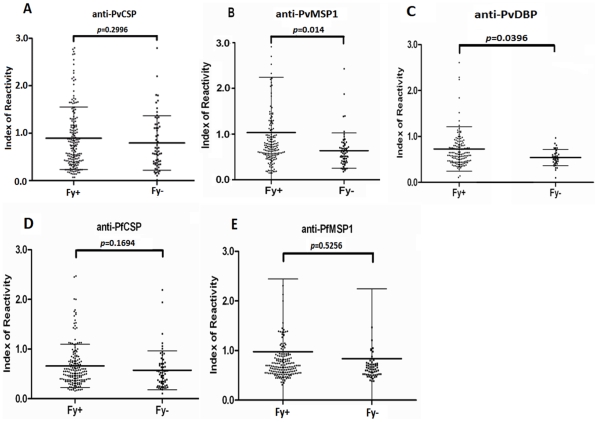
Antibody responses against *P. vivax* erythrocytic antigens are significantly greater in Fy+ than in Fy− individuals. Immune recognition of *P. vivax* sporozoite antigen (A: PvCSP) and blood stage antigens (B: PvMSP1, C: PvDBP), or *P. falciparum* sporozoite antigen (D: PfCSP) and blood stage antigen (E: PfMSP1) by sera of Fy+) and Fy− donors were assessed by ELISA. Values are expressed as Index of Reactivity (IR) that is calculated as test OD divided by cut-off OD. The cut-off OD is the average OD value of US donors (with no prior exposure to malaria)+3 SD.

About a third of the donors living in malaria-endemic regions of Apartado and Turbo had significant antibodies against PvCSP (n = 80, 34%). The majority of those that had PvCSP antibodies were Fy+ (n = 60, 75%) and the remaining were Fy− (n = 20, 25%). The frequency of responses was however comparable between Fy+ and Fy− (35% and 33%, *p* = 0.85) (**Table 2**). The magnitude of responses measured by IR was also similar between Fy+ and Fy− populations (0.88 and 0.79 respectively, *p* = 0.2996) (**Figure 2A**).

### Antibodies against *P. vivax* erythrocytic antigens differ between Duffy positive and Duffy negative donors

A total of 49 donors (25%) living in the malaria-endemic areas had significant antibodies against PvMSP1. The majority of those with PvMSP1 antibodies (90%) were Fy+ (n = 44). The frequency of the responses was significantly higher in Fy+ (16%) than in Fy− (8%) donors (*p* = 0.005) (**Table 2**). The magnitude of the responses (IR distribution) was also significantly different between Fy+ and Fy− donors (*p* = 0.014) (**Figure 2B**). Furthermore, of the 185 samples from malaria-endemic regions assessed, only 17 (9%) had antibodies against PvDBP and all were Fy+ donors (17 out of 147 tested, 12%). No Fy− donor from malaria-endemic regions had antibodies against PvDBP. Therefore, both the frequency and magnitude of the responses against PvDBP were significantly different between Fy+ and Fy− donors (*p* = 0.001 for frequency and *p* = 0.0396 for IR, respectively) (**Table 2** and **Figure 2C**).

In contrast, the frequencies and magnitudes of antibodies against both *P. falciparum* sporozoite antigen (PfCSP) and erythrocytic antigens (PfMSP1) were comparable among Fy+ and Fy− donors. Only 14% (n = 33) of donors living in malaria-endemic areas had antibodies against PfCSP. There were no significant differences in terms of the frequencies (*p* = 0.308) and magnitude (*p* = 0.1694) of the responses between Fy+ and Fy− donors (**Table 2** and **Figure 2D**). Among 36 donors (15%) who had antibodies against PfMSP1, there was also no difference in the frequencies (*p* = 0.175) and magnitude (*p* = 0.5265) of the responses (**Table 2** and **Figure 2E**).

### Frequency and magnitude of antibodies against *P. vivax* erythrocytic antigens in Duffy positive donors varies with FY genotypes

**Figure 3:**
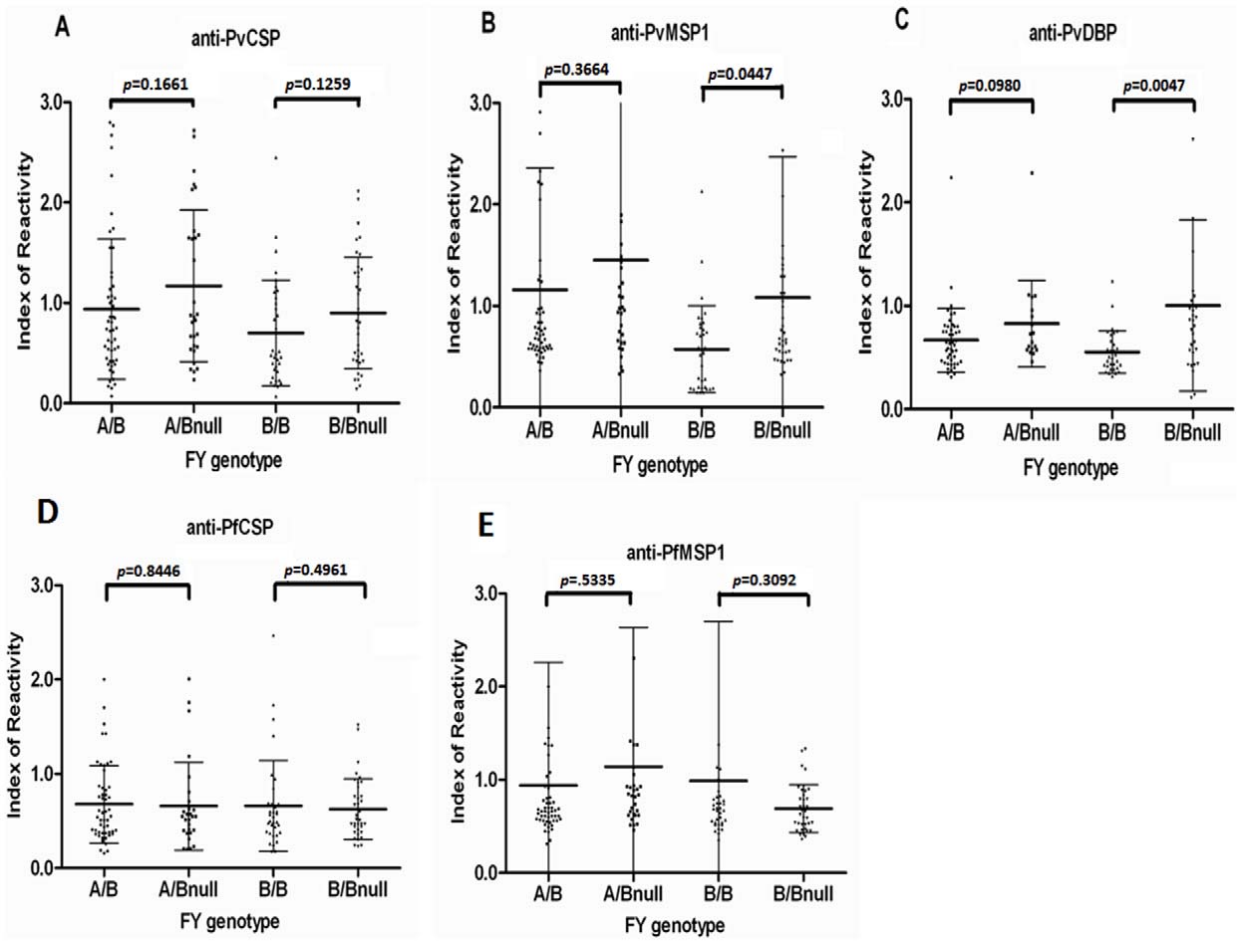
The levels of antibody responses against *P. vivax* erythrocytic antigens differ among different Duffy genotypes. Immune recognition of *P. vivax* sporozoite antigen (A: PvCSP) and blood stage antigens (B: PvMSP1, C: PvDBP), or *P. falciparum* sporozoite antigen (D: PfCSP) and blood stage antigen (E: PfMSP1) by sera of donors with different Duffy genotypes FY*A/FY*B (A/B), FY*A/FY*Bnull (A/Bnull), FY*B/FY*B (B/B) and FY*B/FY*Bnull (B/Bnull). Values are expressed as Index of Reactivity (IR) that is calculated as test OD divided by cut-off OD. The cut-off OD is the average OD value of US donors (with no prior exposure to malaria)+3 SD. The IR of donors with FY*A/FY*B genotype is compared with those of FY*A/FY*Bnull, and FY*B/FY*B with FY*B/FY*Bnull using Student's *t*-test and *p* values<0.05 were considered significant.

Among the specific Fy+ genotypes, the frequencies of responses against PvCSP among those with double positive alleles (FY*A/FY*B and FY*B/FY*B) were low compared to their corresponding single negative allele genotypes (FY*A/FY*Bnull, FY*B/FY*Bnull). 37% and 26% compared to 43% and 42%, respectively (**Table 2**). However, the differences were not statistically significant (**Table 2**, **Figure 3A**).

Unlike PvCSP, DARC is required for erythroctic vivax invasion. We compared the antibodies to blood stage antigens with the stratified DARC expression based on (FY*A/FY*A or FY*A/FY*B)>(FY*A/FY*Bnull, FY*B/FY*Bnull, or FY*B/FY*B) [29]. The antibody responses to PvMSP1 were detected in sera from 16 out of 73 in the high DARC expression group (FY*A/FY*A and FY*A/FY*B) compared to 28 out of 100 in the low DARC expression group (FY*A/FY*Bnull, FY*B/FY*Bnull, or FY*B/FY*B). The difference was not significant between the two groups (*p* = 0.364). However, there were significant differences in the prevalence of antibodies to PvDBP between the high and the low DARC expression groups (3/73 vs. 14/100, respectively) (*p* = 0.031). This may be the first hint that high DARC expression may be associated with low antibody responses to vivax blood stage antigens.

We next wanted to determine which specific genotypes of DARC may be responsible for antibody responses. For PvMSP1, the frequency of the responses was much higher in FY*B/FY*Bnull (12/36, 33%) than in FY*B/FY*B (3/34, 9%) (*p* = 0.012) individuals (**Table 2**). In addition, the magnitude of the responses was also significantly greater in FY*B/FY*Bnull (IR = 1.08±0.58) than in FY*B/FY*B (IR = 0.57±0.43) (*p* = 0.0447) (**Table 2** and **Figure 3B**). A similar low trend in the responses to PvMSP1 was also observed in FY*A/FY*B compared to that in FY*A/FY*Bnull (25% and 43%, respectively). However, the differences in terms of frequency *(p* = 0.086) and magnitude (*p* = 0.3664) of the responses in this paired group were not significant (**Table 2** and **Figure 3B**).

For PvDBP, the frequencies of the responses were found obviously higher among those with a single negative FY allele (22% for FY*A/FY*Bnull and 27% for FY*B/FY*Bnull) than in those with corresponding double positive FY alleles (4% for FY*A/FY*B and 6% for FY*B/FY*B) with *p* = 0.025 and 0.022, respectively (**Table 2**). However, the magnitudes of the responses are different only between FY*B/FY*B (IR = 0.55±0.21) and FY*B/FY*Bnull (IR = 1.04±0.84) (*p* = 0.0047) (**Table 2**), but not between FY*A/FY*B and FY*A/FY*Bnull (*p* = 0.098) (**Figure 3C**).

However, with *P. falciparum* sporozoite (PfCSP) and erythrocytic antigens (PfMSP1), no such trend was observed among the specific Fy+ genotypes (**Table 2**). The frequencies of the responses against PfCSP in FY*A/FY*B and FY*A/FY*Bnull donors were 21% and 14%, respectively, while those in FY*B/FY*B and FY*B/FY*Bnull were 12% and 11%, respectively (**Table 2**). There were also no significant differences in the magnitudes of the responses in these paired groups measured by IR distribution (*p* = 0.8446 and 0.4961, respectively) (**Figure 3D**). For PfMSP1, the frequencies of recognition by FY*A/FY*B and FY*A/FY*Bnull were 19% and 23% respectively, while those for FY*B/FY*B and FY*B/FY*Bnull were 15% and 11%, respectively (**Table 2**). The IR distribution was also similar between both groups (*p* = 0.5335 and 0.3092, respectively) (**Figure 3E**).

### IgM and IgG subclass profiles in reactivity to *P. vivax* erythrocytic antigens

**Figure 4:**
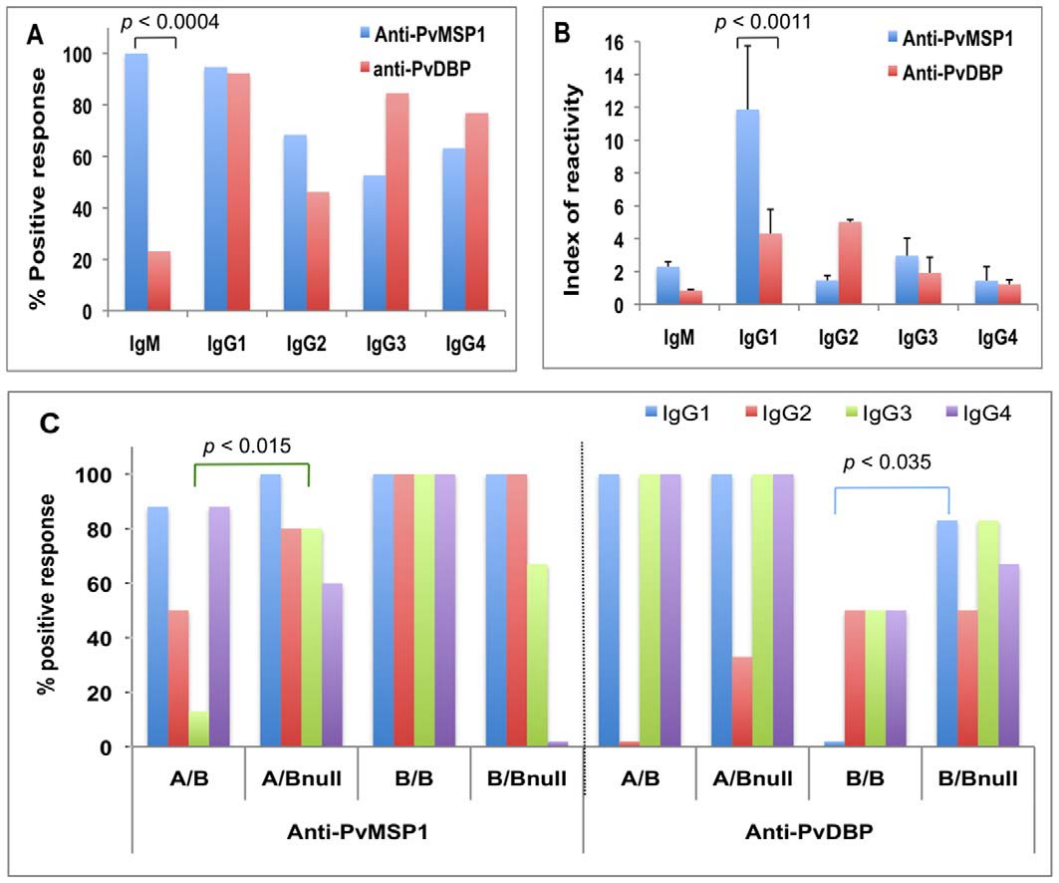
*P. vivax* erythrocytic antigen specific IgM and IgG subclass profiles among different Duffy genotypes. IgM and IgG subclass among Fy+ donors that had total IgG against PvMSP1 and PvDBP was measured by ELISA to determine the frequencies (A) and magnitude (B) of responses against PvMSP1 and PvDBP. IgM and IgG subclass against PvMSP1 and PvDBP were also compared among donors with different Duffy genotypes (C). Samples are considered positive when the OD reading is > average OD of naïve controls + 3SD for frequency of responses or index of reactivity (IR) is greater than 1 for the magnitude of responses, respectively.

To search for which subtype(s) of immunoglobulin (Ig) may be responsible for the antibody responses to erythrocytic antigens, we analyzed the IgM and IgG subclasses within 29 Fy+ donors who had high magnitudes of total IgG antibodies against PvMSP1 (17 donors) and/or PvDBP (12 donors) (**Figure 4**). One hundred percent of donors tested had IgM to PvMSP1, whereas, only 23% of the donors who had IgG to PvDBP had detectable IgM to PvDBP. The difference in frequencies of IgM responses to PvMSP1 versus PvDBP is significant (p<0.0004) (**Figure 4A**). The majority of Fy+ donors that responded to PvMSP1 had IgG1 (95%). The frequencies of other IgG subclasses to PvMSP1 were at similar levels (IgG2 at 68%, IgG3 at 53%, and IgG4 at 63%, respectively). The dominant subclass IgG to PvDBP was IgG1 (92%), then IgG3 (85%), IgG2 (77%), and IgG4 (46%), respectively (**Figure 4A**). The magnitude of IgG1 (IR = 11.9±3.37) was also dominating the responses to PvMSP1, and then IgG3 (IR = 2.96±1.07), IgG2 (IR = 1.47±0.15), and IgG4 (IR = 1.45±0.27), respectively (**Figure 4B**). The magnitude of IgG1 to PvDBP was significantly lower than that to PvMSP1 (IR at 4.3 versus 11.9, *p*<0.0011). However, there were no statistically significant differences in terms of the magnitudes of other IgG subclasses between PvDBP and PvMSP1 (**Figure 4B**).

We further compared the frequencies of the IgG subclasses to PvMSP1 and PvDBP among the different FY genotypes. Significant differences were detected in the frequencies of IgG3 responses to PvMSP1 between FY*A/FY*B and FY*A/FY*Bnull (*p* = 0.015), and of IgG1 to PvDBP between FY*B/FY*B and FY*B/FY*Bnull (*p* = 0.035). There was no additional pattern observed for other IgG subclasses to PvMSP1 and PvDBP (**Figure 4C**).

## Discussion

The lack of DARC among almost all individuals of West African origin has been suggested to be due to natural selection induced by *P. vivax* [56], [57]. This idea, however, is controversial and *P. vivax* has been observed in some populations lacking DARC, indicating that the parasite may be able to use other host cell receptors for invasion if DARC is not present [28], [58], [59].

The presence or absence of DARC has been associated with resistance and susceptibility to a range of infectious and non-infectious diseases [13], [14], [22], [23], [24], [25]. Individuals that lack DARC (Duffy negative, Fy−) are known to be ‘resistant’ to *P. vivax* erythrocytic infection [13], [14]. They are, however, still susceptible to the sporozoite-induced liver stage infection for which DARC is not required. Our earlier study and those of others revealed that humoral and cellular immune responses to *P. vivax* pre-erythrocytic antigens such as CSP are present in Fy− individuals [31], [32]. Among Fy+ individuals, however, the influence of each allele on the acquisition of immune response against vivax antigens has yet to be fully explored.

The expression levels of erythroid-specific DARC varied with erythrocyte age and between different FY+ genotypes [43], [60], [61]. DARC expression was 2-fold higher in FY*A/FY*A homozygotes than in FY*A/FY*Anull heterozygotes among people living in Papua New Guinea, and higher DARC expression was associated with the higher prevalence of *P. vivax* infection seen in FY*A/FY*A compared to FY*A/FY*Anull subjects [43]. This was caused by the significant reduction of the adherence of Duffy binding protein to the erythrocyte in individuals who carried the FY*A/FY*Anull allele [60]. Individuals with the FY*B/FY*B genotype had a higher risk of *P. vivax* infection than those with FY*B/FY*Bnull genotype in malaria-endemic regions of Brazil [44]. Moreover, DARC expression has been found to be lower in the FY*B/FY*B than in FY*A/FY*A and FY*A/FY*B genotypes [61]. Hence, apart from the different levels of FY expression, the specific qualities and subsequent functions of the DARC upon binding to the *vivax* Duffy binding protein and post invasion of erythrocytes, as well as its role in innate immunity as a receptor for chemokines may all contribute to the resistance and susceptibility to vivax infection. We were motivated to investigate the influence of different Duffy positive alleles on the acquisition of anti-*P. vivax* immunity among individuals living in malaria-endemic regions.

First, we demonstrated that broad antibody responses were detected by ELISA to both *P. vivax* and *P. falciparum* in individuals living in the two malaria endemic areas in Colombia that we studied (**Figure 1**). The higher prevalence of recognition of at least one *P. vivax* antigen (44%, n = 103) compared to recognition of the corresponding *P. falciparum* antigen(s) (27%, n = 64) probably reflects the higher level of *P. vivax* transmission in these areas compared to that of *P. falciparum* [62], [63]. However, the overall low prevalence of recognition of the individual antigens reflects the relatively low level of malaria transmission in these areas and the fact that our selection criteria excluded individuals with active infections.

The high level of recognition of PvCSP regardless of the presence or absence of DARC had previously been recorded among individuals living on the Pacific coast of Colombia [32] and in Brazil [64]. There was no significant difference in the frequency of antibodies to the sporozoite and liver-stage antigen PvCSP among Fy+ and Fy− individuals (*p* = 0.2996). Since sporozoites do not use DARC to infect hepatocytes, Fy+ and Fy− individuals should be equally susceptible to sporozoite infection [65], [66].

To determine whether the acquired antibody responses associated with “natural” protection, we focused our studies on donors that had developed acquired antibody responses but who had undetectable parasitemia by blood smear at the time of enrollment and no history of malaria symptoms in the past 12 months. Long-lasting antibodies and memory B cell responses in other low malaria transmission endemic areas have also been reported to be associated with naturally acquired protection [35]. As we show in **Table 2**, the antibody responses to PvMSP1 differed significantly between Fy+ and Fy− individuals in both frequency (*p* = 0.005) and magnitude (*p* = 0.014) (**Figure 2B**). This observation is consistent with the requirement for DARC for *P. vivax* erythrocytic infection [31], [32]. We suspect that the very few Fy− donors in the current study that did have detectable anti-PvMSP1 antibodies had been transiently exposed to PvMSP1 after the release of merozoites from infected hepatocytes into the blood. These merozoites would have been cleared rapidly from the circulation because they would be unable to invade the Fy− erythrocytes in these individuals.

The major finding in this study was within the Fy+ group (**Table 2** **and** **Figure 2** **–** **3**), in which the frequencies and magnitudes of the antibody responses to *P. vivax* erythrocytic antigens were significantly higher in individuals possessing a single negative allele (FY*A/FY*Bnull and FY*B/FY*Bnull) than in double positives (FY*A/FY*B and FY*B/FY*B). This is exactly opposite of the expected level of DARC expression [43], [61] and susceptibility to *P. vivax* infection [42], [67]. It is known that the elevated DARC expression in double-positive individuals confers a higher risk of *P. vivax* infection in comparison to those with one negative gene (FY*A/FY*Bnull, FY*B/FY*Bnull) [43], [44], [60], [61]. Furthermore, active erythrocytic malaria infections have been reported to induce immune suppression that prevents the host from mounting an effective immune response against the blood stage parasites and other co-infecting agents [38], [68], [69], [70], [71], [72], [73]. This immune suppression ranges from inhibition of dendritic cell maturation [38], to inhibition of the generation of specific CD4 T cells [69] and apoptosis of specific CD4 T cells [73]. Thus, it is likely that the high susceptibility to *P. vivax* blood stage infection and concomitant high *P. vivax* erythrocytic parasite load in FY*A/FY*B and FY*B/FY*B double-positive individuals may contribute to the suppression of antibody responses against erythrocytic antigens when compared with FY*A/FY*Bnull and FY*B/FY*Bnull individuals. The higher antibody levels observed in those with a single negative FY allele may also limit parasite load during subsequent infections that may in turn reduce or prevent the immune suppression induced by erythrocytic parasites. Finally, based on DARC's role as a sink for excess pro-inflammatory cytokines [74], high levels of DARC expression in FY double-positives may reduce the surplus of pro-inflammatory cytokines and curb the severity of symptoms; alternatively, DARC may down-regulate the immune responses that control the erythrocytic parasitemia.

This difference in immune recognition between FY*A/FY*B and FY*B/FY*B individuals and FY*A/FY*Bnull and FY*B/FY*Bnull individuals observed with PvMSP1 was confirmed with a second *P. vivax* blood stage antigen, PvDBP, which is involved in *P. vivax* erythrocytic invasion. Our observations that the antibody responses to two *P. vivax* blood stage antigens are higher in single positive than in double-positive individuals leads us to speculate that immune responses to multiple erythrocytic antigens in hosts with low parasite load could act synergistically against erythrocytic parasite invasion and development, providing clinical protection against subsequent reinfection.

In order to determine whether the differential frequencies of recognition to blood versus sporozoite antigens by FY genotypes were restricted to *P. vivax*, we investigated the recognition of PfCSP and PfMSP1 in the same study population (**Table 2**, **Figures 2** **and** **3**). As expected, no such variation in antibody response against *P. falciparum* antigens with FY genotype was observed.

Finally, we wanted to know whether any specific Ig subtypes are responsible for the naturally acquired antibodies that are associated with individual DARC genotypes. IgM responses to malaria are more likely to be detected early after infection and are expected to switch from the IgM isotype to the cytophilic isotypes IgG1 and/or IgG3, a switch that has been associated with clinical control of erythrocytic parasites [75], [76]. In some endemic areas the IgG1 response to PvMSP1 is higher, whereas in other areas the IgG3 response dominates [77]. In this study, strong IgM and IgG responses to PvMSP1 were detected in the same Fy+ individuals (**Figure 4**), indicating that the IgM response did not compromise the induction and development of IgG1 and IgG3 responses to this antigen. The magnitude of IgG1 response to PvMSP1 was 3 times greater than the IgG1 response to PvDBP. However, the frequency of IgG3 responses to PvMSP1 was significantly lower in FY*A/FY*B than in FY*A/FY*Bnull (*p* = 0.015) individuals, as were the IgG1 responses to PvDBP in FY*B/FY*B compared to FY*B/FY*Bnull individuals (*p* = 0.035). Therefore, the lower IgG3 and IgG1 components of the total IgG response may account for the decreased responses to *P. vivax* erythrocytic antigens in humans with the double positive FY*A/FY*B and FY*B/FY*B genotypes, respectively (**Figure 4C**). However, the immune mechanism by which differential DARC expression manipulates the specific IgM and IgG subclass profiles associated with clinical protection need to be determined.

In summary, we observed that frequency and magnitude of antibodies specific for *P. vivax* erythrocytic antigens varied with the host DARC genotype. Donors with genotypes associated with higher levels of DARC expression and higher susceptibility to *P. vivax* infection were found to have lesser frequencies and lower magnitudes of specific antibodies against PvMSP1 and PvDBP. IgG3 and IgG1 may account for the decreased responses to *P. vivax* erythrocytic antigens in humans with FY*A/FY*B or FY*B/FY*B genotypes. This supports the notion that one of the primary mechanisms by which *P. vivax* evades host immunity is through DARC indirectly down-regulating humoral responses against erythrocytic invasion and development. These results represent an important advance in our understanding of blood-stage immunity to *P. vivax* that will inform the rational design and development of effective vaccines to control *P. vivax* malaria.

## Materials and Methods

### Study populations

Subjects were recruited from two malaria-endemic cities of Turbo (8°5′42″N, 76°44′123″W) and Apartado (7°52′40″N, 76°37′44″W) both of which are on the Caribbean coast of Colombia, South America. Their inhabitants are mainly a mixture of indigenous, Hispanic and African backgrounds. Banana cultivation is the major source of income. They both have high migration rates, and malaria transmission is perennial and unstable, with a mean of 10 infectious bites per 1,000 people. During the 2004 to 2008 period, the means of annual *P. vivax* infectious bites are 323 in Turbo and 84 in Apartado per 1,000 inhabitants. *P. falciparum* is less frequent in these two cities during the same period (mean annual infectious bites: Turbo = 44, Apartado = 11) (personal communication with local health authorities) [62], [63], [78]. The causes of malaria infections in these two areas are comparable; 72% vivax, 28% falciparum and only 0.11% with mixed infection. All the subjects were above 18 years old and were voluntarily recruited. The purpose of the study was explained to each subject, after which they signed the consent form to participate in the study. Only subjects who had long been residing in the study area for more than 5 years and have no symptoms of malaria within the past year were enlisted. Subjects included in the study were only those with no detectable *P. vivax* or *P. falciparum* parasites in blood smears on the day of sample collection. A total of 233 subjects were enrolled from malaria-endemic areas of Turbo (n = 155) and Apartado (n = 78) (**Table 1**). For malaria infection naïve controls with specific DARC genotypes, 30 subjects were recruited from Medellin, where there is no malaria transmission. Additional eight adult US donors who had no previous history of malaria or trip to malaria-endemic region were also used as ELISA assay control. Whole blood samples were obtained for Duffy genotyping and the separated sera were used for anti-malaria antibody screening. The study protocol and consent forms were approved by the Ethics committee for research in humans of Sede de Investigacion Universitaria (CBEIH-SIU), Universidad de Antioquia (Medellín, Colombia) and Western Institute Review Board (WIRB), USA.

### Duffy genotyping

Genomic DNAs from the peripheral blood of all donors were purified using the QIAamp kit (Qiagen), and the quality was assessed in 1.5% agarose gel. PCR was performed to amplify 1000 bp using the following primers: P1, 5′CCTTTTTCCTGAGTGTAGT3′ (sense) and P2, 5′GCAGAGCTGCCAGCGGAAGA3′ (antisense) as described previously (68). The PCR conditions were: 95°C 4 min, 35 cycles of 1 min at 94°C, 1 min at 58°C, and 1 min at 58°C, and final 10 min at 72°C. PCR products were purified using Qiagen kit (Valencia, CA) for sequence analysis. Primers P1, P2, or P38 (5′AGGCTTGTGCAGGCAGTG3′) (68) were used for sequencing reactions. A single nucleotide substitution (T46C) at the promoter region abolishes the expression of the DARC in erythroid tissues (Fy^−^, genotype FY*Bnull/FY*Bnull, phenotype Fya^−^b^−^). A single nucleotide substitution (G131A) at the exon region of DARC determines the allele specificity among Duffy positive donors (Fy^+^).

### Recombinant antigens

The recombinant *P. vivax* circumsporozoite protein (PvCSP) that includes the amino terminus repeat region (3X repeat 1 and 3X repeat 2), and carboxyl terminus was provided by Dr. KL Sim (Protein Potential LIc, MD). Recombinant *P. vivax* MSP1 corresponding to MSP1 *P. falciparum* merozoite surface protein 1 (PfMSP1_19_) and *P. falciparum* MSP1 were obtained from MR4, VA. Recombinant *P. vivax* Duffy Binding Protein (PvDBP) corresponding to region II of the protein was kindly provided by J.H Adams (University of South Florida, Tampa, FL) and *P. falciparum* CSP long synthetic peptide (LSP) corresponding to the amino acid 282–383 of the C-terminal region was obtained from Dr. G. Corradin (University of Lausanne, Switzerland) [79].

### Evaluation of antibody responses

Enzyme Linked ImmunoSorbent Assay (ELISA) was performed in 96 well flat-bottom-plates (Nunc-Immuno Module, USA). Wells were coated with recombinant proteins PvCSP, PvMSP1 or PfMSP1 at 2.5 µg/mL (PvDBP at 2 µg/mL, PfCSP LSP at 1 µg/mL) in 50 µl of PBS buffer (pH 7.4) and incubated overnight at 4^0^C. The plates were washed with 200 µl of wash buffer (0.05%Tween-20 in PBS) using Bio-TEK Washer (ELx405) and then blocked with 200 µl of blocking buffer (5% nonfat dry milk in PBS) for 1 hour at room temperature (RT). Fifty microliters (50µl) of 1∶50 serum dilutions were added to the coated wells and then incubated for 2 hours at room temperature. After another washing, 50 µl of HRP-labeled goat anti-human IgG (H+L) at 1∶4000 in 2.5% milk PBS-Tween-20 was added and the plates were kept at room temperature for 1 hour. For assessment of IgM and IgG1-4 subtypes, the HRP-labeled mouse anti-human antibodies were used at 1∶1000 following the manufacturer's instruction (Southern Biotech, Birmingham, AL, USA). They were later developed with tetramethylbenzidine (TMB) substrate (Sigma-Aldrich) and the OD was measured at 450 nm with ELISA Autoreader MR5000 (Dynatech, Chantilly, CA). All the assays were performed in triplicates and repeated twice. The antibody recognition by individual sera is expressed as Index of Reactivity (IR) that is test OD reading divided by cut-off OD. The cut-off OD is calculated as the average OD reading of negative control donors (US donors with no prior exposure to malaria)+3 standard deviations. A sample is considered positive when the IR value is ≥1.

### Statistical analysis

Statistical analyses were performed using Graphpad prism 5. Differences in the means were assessed using Student's *t*-test and Chi-squired test. *P* values <0.05 were considered significant.
